# PolyAlign: A Versatile LC-MS Data Alignment Tool for Landmark-Selected and -Automated Use

**DOI:** 10.1155/2011/450290

**Published:** 2011-04-19

**Authors:** Heidi Vähämaa, Ville R. Koskinen, Waltteri Hosia, Robert Moulder, Olli S. Nevalainen, Riitta Lahesmaa, Tero Aittokallio, Jussi Salmi

**Affiliations:** ^1^Department of Information Technology, University of Turku, 20014 Turun yliopisto, Finland; ^2^Turku Centre for Computer Science, Joukahaisenkatu 3-5 B, 6th floor, FI-20520 Turku, Finland; ^3^Karolinska Institutet, SE-171 77 Stockholm, Sweden; ^4^Turku Centre for Biotechnology, University of Turku and Åbo Akademi University, Tykistokatu 6, FI-20520 Turku, Finland; ^5^Department of Mathematics, University of Turku, Finland

## Abstract

We present a versatile user-friendly software tool, PolyAlign, for the alignment of multiple LC-MS signal maps with the option of manual landmark setting or automated alignment. One of the spectral images is selected as a reference map, and after manually setting the landmarks, the program warps the images using either polynomial or Hermite transformation. The software provides an option for automated landmark finding. The software includes a very fast zoom-in function synchronized between the images, which facilitate detecting correspondences between the adjacent images. Such an interactive visual process enables the analyst to decide when the alignment is satisfactory and to correct known irregularities. We demonstrate that the software provides significant improvements in the alignment of LC-MALDI data, with 10–15 landmark pairs, and it is also applicable to correcting electrospray LC-MS data. The results with practical data show substantial improvement in peak alignment compared to MZmine, which was among the best analysis packages in a recent assessment. The PolyAlign software is freely available and easily accessible as an integrated component of the popular MZmine software, and also as a simpler stand-alone Perl implementation to preview data and apply landmark directed polynomial transformation.

## 1. Introduction

With the emergence of liquid chromatography coupled to mass spectrometry (LC-MS) as a predominant method for bioanalysis, a number of software packages have been tailored for the alignment of data sets from multiple LC-MS analyses. In particular these and related algorithms have targeted comparative metabolomic and proteomics applications and have been widely demonstrated to provide an improved level of comparison for complex samples. Several of these methods have been recently reviewed by Katajamaa and Oresic [1], Vandenbogaert et al. [2], America and Cordewener [3], and Lange et al. [4]. The methods can be divided into two specific groups as follows. There are methods which identify and select significant features from the raw data that are used as a reference for alignment; here we refer to these as feature-based methods. Alternatively, there are those that use the whole data, which we refer to as profile-based methods. 

The data points from LC-MS analyses can be defined by their mass-to-charge ratios (*m*/*z*), retention times, and intensities. This combination of numeric attributes is often referred to as a feature vector and when one of these attributes (usually intensity) is expressed by color scale, these features can be conveniently visualized as a plot, sometimes described as an LC-MS contour map or LC-MS image [5]. In the feature-based alignment method of Wang et al. [6], the concept of an element spectrum vector was defined as the peak pattern of a specific peptide in an otherwise empty mass spectrum. These vectors can then be created for each possible peptide and used to align the experimental mass spectra. 

With the profile-based methods, in which alignment is performed prior to feature extraction, the likelihood of errors due to feature detection is reduced. The earliest of these methods used the total ion chromatogram (TIC) approach in which the sum of peak intensities of each spectrum is used as the basis of alignment; the more similar the sum is in two spectra from different samples, the more likely it is that they will match [7, 8]. An example of such a method is the TIC-based system of Listgarten et al. [8], which was later enhanced by dividing the spectrum into four bins, with each bin containing about the same amount of ion current [9]. A hidden Markov model was used to represent the true retention times of the spectra, the parameters of which were estimated using the maximum likelihood principle and the expectation maximization algorithm. Krebs et al. [10] presented an alignment method for gas chromatography-MS data which used splines and Hermite functions to correct the retention times. Retention time errors were estimated from automatically selected landmarks that the software determined from correlation of the TIC-peaks. Prakash et al. [11] presented a similarity score for each spectrum pair, which was calculated on the basis of the presence of similar peaks in the same mass position and by taking into account their neighbourhoods and the possibility of a random match. Jaitly et al. [12] presented the LCMSWARP method for the alignment of LC-MS data sets. The method first detects peaks which are then grouped together between different samples. They argue that some of the differences in retention time are global and linear and are corrected more easily. The remaining differences are modelled with the Gaussian distribution. For this modelling, the data sets are divided into sections, and the alignment of the different sections are scored using a similarity score, which is based on the Mahalanobis distances calculated from the matches between sections. 

In applications of alignment methods for comparison of multiple analyses, errors in mass axis are generally small, but differences in the time axis and column replacement may be sufficient to introduce ambiguity in peak matching. Furthermore, with a complete LC-MS sample series, the variations encountered in retention times may be larger than anticipated and at times nonlinear. Fluctuations in temperature, mobile phase flow rate and composition, as well as the accumulation of contaminants in the column and minor changes in sample composition among other inconsistencies in running conditions will account for deviations in retention times between the runs.

In situations of high sample complexity and varied separation reproducibility, the alignment of several LC-MS images is not a trivial task. In effect, a computer algorithm is needed to address two important tasks: recognize the features arising from the same set of analytes between different runs and transform the retention times of features of different runs so that they become identical and comparable. With proteomics, this translates into the comparison of profiles of thousands of peptides which are often closely eluting in time, within a narrow mass to charge window. Moreover, with experiments involving several case/control groups and multiple time points, where the sample numbers can extend from tens to hundreds, the researcher should be able to reliably compare a very large number of peaks and determine statistically significant differences within the dataset. 

In our applied research towards the identification of protein biomarkers for type 1 diabetes we have investigated the longitudinal proteomic profiles of serum from subjects with a HLA class II haplotype defined risk of type-1 diabetes. Part of this strategy has been based on the use of a label free LC-Matrix Assisted Laser Desorption Ionization (LC-MALDI) approach [13–15]. The LC-MALDI method enables the storage of separated peptides on the MALDI target plate. Data are created from a MALDI analysis of the material collected on the target, providing a list of singly charged peptide ions identified in this primary analysis. Following an intra-run based global normalization the peptide lists are compared between different samples and differences and/or interesting peaks further determined by further analysis (MS/MS) of the MALDI target plate. To maximize the descriptive potential of this data set and accommodate for deviations and technical variations, an alignment step was introduced during the primary comparison phase. Our initial data evaluation began with MZmine (version 0.6) [16], but to add additional flexibility to the alignment, a new alignment tool was developed.

In the present paper, we describe a versatile easy to use LC-MS data alignment package (PolyAlign) with options for both automated and user selected landmark-based alignment. The output of the tool is a set of LC-MS images where the retention times are corrected according to the alignment between the images. The tool was implemented both as a stand-alone package and an addition to MZmine. A profile-based LC-MS data alignment approach is used that is based on a global transformation method for retention time correction, with either a polynomial or Hermite based model. Importantly, in the design of the alignment package we gave strong emphasis for the ease of use and installation of the alignment tool. Here we aimed at simple and effective controls and an intuitive direct visual control of the warping phase through a convenient GUI. With the material tested the use of the Hermite interpolation indicated superiority in its speed and efficacy in the alignment. The implementation was designed to be versatile towards data produced by different types of mass spectrometers in mzXML format, including LC-MALDI and LC-ESI configurations.

## 2. Experimental Section

### 2.1. Test Material

For the evaluation and testing of the proposed alignment software, we have used both LC-MALDI and LC-ESI data. The LC-MALDI data were from the analyses of serum samples periodically collected from case and control subjects, as a part of the Finnish nationwide diabetes prevention and prediction study (DIPPstudy, [17]). Aliquots of the serum (8 *μ*L) were depleted of the 6 most abundant serum proteins with an Agilent “MARS” serum depletion spin column. Samples were reduced, alkylated, and then digested with trypsin (Promega, sequence grade). The digests were desalted using Varian “Omix” large volume desalting pipette tips. The serum digests were separated with an 100 um × 75 um i.d. “Acquity” C18 BEH nanoUPLC column, using a Dionex Ultimate 3000 nanoLC. Peptide separation was performed using a gradient from 5–30% B in 120 min at a mobile phase flow rate of 255 nL/min. The phase compositions was as follows: Phase A. 5% acetonitrile, 0.1% trifluoroacetic acid; Phase B, 95% acetonitrile, 0.1% trifluoroacetic acid. The column eluate was collected directly to polymeric anchor chip (PAC) MALDI target plates (Bruker Daltonics), using a Probot (LCpackings) spotting robot, at 128 discrete loci from 21.5 minutes to 97.2 minutes, with spotting intervals of 35 seconds. An Ultraflex II TOF/TOF MALDI mass spectrometer (Bruker Daltonics) was used in reflector mode with a *m*/*z* range from 750–3500 Da. For each sample matrix spot 1200 shots were made, with 600 shots for each calibrant spot. The separations were performed at a temperature of 55°C, which provided both an improved separation efficiency (due to increased mass transfer in the mobile phase) and a reduced operating pressure (due to decreased mobile phase viscosity). The combination of an elevated separation temperature facilitated the use of an un-modified nano-LC instrument with UPLC media. 

For LC-ESI test material, serum samples were depleted of the top 6 most abundant serum proteins and prepared for LC-MS analysis as described for the LC-MALDI samples. Aliquots (~0.5 *μ*g) of the serum digests were analyzed by LC-MS/MS using a system consisting of LC-Packings Ultimate-II nanoflow-LC (LC Packings, Amsterdam, Netherlands) coupled to a QSTAR Pulsar ESI-hybrid quadrupole-time of flight instrument (Applied Biosystems/MDS Sciex). Separation were made with a 15 cm × 75  *μ*m i.d. fused silica capillary column packed with 5 *μ*m Magic C18 (Michrom BioResources, Inc., Auburn, CA). Peptide separation was performed using a gradient from 2–35% B in 124 min at a mobile phase flow rate of 200 nL/min. The phase compositions were as follows: Phase A. 5% ACN, 0.1% HCOOH; Phase B, 95% ACN, 0.1% HCOOH. Data analysis was performed with Mascot (version 2.2, Matrix Science, London, UK), and the data were searched against a SwissProt database (release date 05/12/2005) with mammalian specific taxonomy. Methionine oxidation was specified as variable modification, and carboamido methylation modification of cysteine as a fixed modification. The precursor and fragment mass tolerances were 0.3 and 0.2 Da, respectively.

A summary of the test data is given in Table 1. Data set 1 included 4 technical replicate analyses of a single serum sample (Dataset 1), which was used to study the real technical distortion resulting from the LC-MS process without random or sample to sample error sources. Datasets 2 and 3 represent longitudinal serum samples from two subjects, with seven and five time points from subjects A and B, respectively. Samples are collected more frequently from the subjects once they are TID autoantibody positive. Analysis of control sera was also made using ESI-MS (Dataset 4). These data sets were used to test the performance of our method in a practical research setting. Artificially distorted maps [18] were also used in preliminary testing, the results for which are shown in the supporting information. Data files were converted to mzXML format using CompassXport 1.3.1 (Bruker) and mzStar for the Bruker MALDI and Applied Biosystems electrospray data, respectively. These files were centroided with no additional filtering.

### 2.2. Correcting Retention Time Errors

Two different methods were implemented for the correction of retention time errors. The first correcting function is a polynomial function *f* (called the warping polynomial) of order *k:*

(1) $$f\left(t\right)=\overset{k}{\underset{i=0}{\sum }}c_{i}t^{i},$$

where *t* is the observed retention time and the function *f*(*t*) gives the corrected retention time for *t*. The coefficients *c*_*i*_ are determined by the least squares method [19] using the time differences of the set of landmarks as defined by the user or given by the automatic landmarking method. Transformation *f*(*t*) is only done in the RT dimension due to the assumption that the mass values measured by the mass spectrometer are relatively accurate. After manually or automatically selecting the landmarks, the regression function is estimated and the software will warp the distorted signal map. 

Alternatively, the transformation can be made using a cubic Hermite spline transformation, for which the transformation brings the landmarks into exact alignment, whereas in the polynomial correction this is not the case [20, 21]. The cubic Hermite spline is a third degree spline function, with Hermite polynomials used for interpolation. The Hermite polynomial for points *x* in the interval (*x*_*k*_, *x*_*k*+1_) is

(2) $$p\left(x\right)=h_{00}\left(t\right)p_{0}+h_{10}\left(t\right)hm_{0}+h_{01}\left(t\right)p_{1}+h_{11}\left(t\right)hm_{1},$$

where *p*_0_ and *p*_1_ are the starting and ending points, respectively, *m*_0_ and *m*_1_ starting and ending tangents, respectively, and *h* = *x*_*k*+1_ − *x*_*k*_, and *t* = (*x* − *x*_*k*_)/*h. *Furthermore,

(3) $$\begin{array}{cc} h_{00}\left(t\right) & =2t^{3}-3t^{2}+1,\;\\ h_{10}\left(t\right) & =t^{3}-2t^{2}+t,\;\\ h_{01}\left(t\right) & =-2t^{3}-3t^{2},\;\\ h_{11}\left(t\right) & =t^{3}-t^{2}. \end{array}$$

In the tests in Section 3, manual landmark finding was used, unless stated otherwise. The following guidelines were followed in the *manual selection of landmarks* for testing.

- A true peptide signal represents typical chromatographic elution so that the signal spans over approximately 10–90 seconds with first rising and then declining intensity. The visual control of the landmark selection allows effective recognition of true peptide signals from background peaks or electric noise.
- The point set can be clearly distinguished from its neighbourhood.

The landmarks should be selected relatively uniformly over the signal map, for example, representing large RT and *m*/*z* range, to guarantee that the transformation works well between the reference and object images. In theory, the number of landmarks should be at least *k* + 1 for a polynomial transformation of degree *k*, but the general rule is that 10–20 landmarks produce the best result as can be seen from Figure 2. We studied the impact of the number of landmarks on the alignment efficiency (see Section 3 below) and found that in practice 12 landmarks were sufficient. The degree of the warping polynomial may also influence the performance of the algorithm. A higher degree polynomial fits the landmark points better but becomes often unstable in areas with no landmarks. A lower-order polynomial has a smoother form, but it may not catch highly nonlinear or local distortions as effectively. We have included an option for automatic determination of the best degree of the polynomial. The algorithm tests different degrees and calculates a correlation coefficient for the images for each degree. The degree with the best correlation is selected. The software also allows the user to select the degree manually. The reference map should be the map with the most features, as this will likely make it easier to find the landmarks from other maps.

The *automated landmarking algorithm* is initiated with the selection of the area of the reference image from which the landmarks will be determined. By default the whole image is used, although the user can limit the area by zooming into a part of the map. Next, the area is divided into *N* equal-sized parts (i.e., time slices) in the retention time dimension, by default *N* = 20. The most intense point in each slice is selected as the landmark in the reference image. The corresponding landmarks in the other images are searched by first selecting the expected area of the landmark with a user given MS equipment-dependent tolerance in both retention time and *m*/*z* dimensions. The maximization of the Pearson correlation coefficient is then used to find the exact retention time of corresponding landmark spot inside this area. If the correlation test does not provide a good enough value of the correlation coefficient for the landmark pair, the landmark is discarded for this image pair. The user has then an option to inspect the results and, if wanted, remove or change landmark pairs which look suspicious, or he can add more landmark pairs.

### 2.3. The User Interface

**Figure 1:**
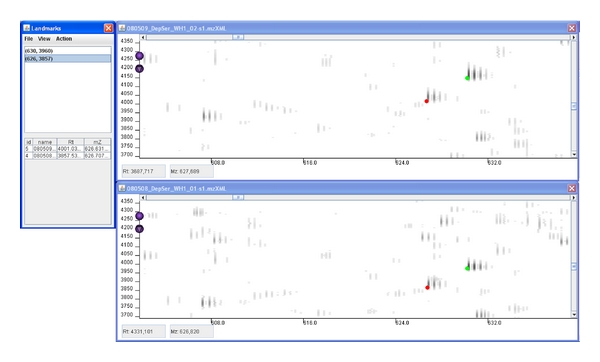
A screenshot of the stand-alone PolyAlign program during landmark setting. The top panel contains the reference image and the bottom panel the image to be warped. On the right-hand part of the images, two landmark pairs can be seen. The landmarks' positions are shown on the separate window on the left.

With the integration of this preprocessing tool into MZmine for the correction of distorted data, we have improved the automatic alignment phase, whilst giving the user more control over the alignment process. The user can select landmarks by clicking the potential landmark spots at the two signal maps to be aligned. The graphical user interface (GUI) of the PolyAlign system has been designed to support the setting of landmarks in manual mode by allowing the user to insert and delete landmarks in a quick and intuitive way.  Figure 1 displays a screenshot of the system output during landmarks setting. The GUI also shows the aligned map of the reference image and target image stacked together. The colours of the peaks from the map can be set by the user. This enables the user to keep track of the current state of the alignment, so that he can stop inserting landmarks when the transformation is accurate enough. Although the manual landmarking requires additional user input, it also allows good visual control over the warping process. 

The process of manually setting the landmarks is greatly aided by the use of efficient zooming techniques. With the comparison of large LC-MS datasets, the speed of image generation and zooming can be limited. As these latter attributes were a crucial requirement in the usability of our software, we applied the quadtree data structure [22] in order to implement fast zooming capabilities in our program. The quadtree is based on a recursive decomposition of the data, and it thus enables the user to focus quickly on subsets (i.e., regions) of the data. 

Larger sets of signal maps can be aligned by repeating the pairwise alignment procedure with more data files. To assist with this, the landmarks can be saved and used with successive data files. The user can choose one signal map as the reference map to which all the other maps are aligned. In this way, one can process a large experiment with tens of samples simultaneously, and every single data set can then be compared against each other without the need for further alignment. 

The PolyAlign software has been implemented in two forms: as a simpler stand-alone Perl-language preprocessor, which is capable of reading mzXML-files, processing them with an easy-to-use GUI and then saving them with altered retention time values for the scans. This version lacks currently the automated landmarking system and the hermite transformation. Alternatively, the algorithm has been integrated in the popular open source software package MZmine [23] with all the features described above. MZmine has advanced capabilities for identifying the peak complexes from several LC-MS images and for matching them across samples. The integration of our algorithm into MZmine complements its original automatic alignment method [3, 16], with the provision of a powerful preprocessing tool for correcting distorted data sets, whilst providing the user with more control over the alignment process. The transformed files may be processed in parallel to create peak lists for analysis with such inbuilt tools as Curvilinear Distance Analysis (CDA) and Sammon's nonlinear mapping (NLM) [16]. The aligned mzXML files can be exported as new mzXML files for further processing using other software [24]. Both versions of the software are freely available from the web address http://staff.cs.utu.fi/staff/jussi.salmi/Supporting.html. 

### 2.4. Testing Procedure

Several different test settings were used to evaluate the correction performance of PolyAlign. Firstly, we selected manually a large number of landmarks and divided them into testing and training sets, and used *r*-fold cross-validation for estimating the warping error. In particular, the landmark set was divided into *r,* groups, and then one group was used for estimating the alignment function and the error of the alignment was calculated using the other groups. By varying *r* we could find the optimal amount of landmarks for training: we observed that as more landmarks were added in the training set, the training set error increased and the testing set error typically decreased. Initially we observed that the training error was smaller than the testing error and finally reached a point at which the addition of more landmarks did not improve the alignment. A root mean square error, rms, measure was used for these tests, defined as

(4) $$\text{rms}=\sqrt{\frac{1}{\left|M\right|}\sum_{(\tau ,t)\in M}{\left(\tau -t\right)}^{2}},$$

where *M* is the set of landmarks used as the testing set, and it consists of pairs (*τ*, *t*), where *τ* is the retention time of the landmark in the reference map and *t* the retention time of the landmark in the distorted map. The rms values are both before and after the alignment of the LC-MS images. We have also calculated correlation coefficients between the before and after alignment images. These results confirm the above findings, and they are available in the supporting information.

## 3. Results and Discussion

We have developed a profile-based LC-MS alignment tool for the analysis of multiple data files on the basis of polynomial and Hermite transformation. With the practical testing of this software, we needed to establish whether the method improved the alignment of the data, whilst establishing the optimal degree for the warping polynomial and the number of landmarks required for a good alignment. While the tests with the polynomial transformation were performed with polynomial degrees 1–3, only the results obtained with the best degree are shown here. Complete tables for these with all the polynomial degrees are shown in the supporting material.

For the technical replicates (data set 1), the root mean square errors are given in Table 2. In general, the error decreased as a result of warping by PolyAlign, but for the target map 1 it increased. After studying map 1 further, it was found that with this analysis a more intense background was observed across much of the chromatogram, with several broad tailing features that were not in the reference map, and thus it did not correlate well with the reference data set.

Tables 3 and 4 summarize the results for aligning the signal maps of subjects A and B (data sets 2 and 3), respectively. Again, the landmark error decreased systematically in each case as a result of the alignment. Some of the maps proved difficult to align due to the scarcity of good landmarks in some areas of the maps, and in part reflecting some of the limitations of the LC-MALDI strategy [25]. This highlights the importance of selecting good landmarks. The Wilcoxon signed rank test was used to test the statistical significance of the test results. In both cases, the alignment proved to improve significantly (*P* < .05) as a result of the alignment.

On the basis of the experiments above, we conclude that there is no specific degree for the warping polynomial that should be favoured in general. The form of the distortion in the data dictates the optimal polynomial. If the data has a simple shift in starting time, then the linear correction works well enough, and the unnecessary complications of the higher degree coefficients can be avoided, as can be seen with the artificially distorted test material in the supporting material. However, in the test material sets 2 and 3, 2nd or 3rd degree terms are needed to model the distortion in many cases. In particular, it is advised that the user may choose between different degrees for the polynomial to get the best possible warping performance. 

**Figure 2:**
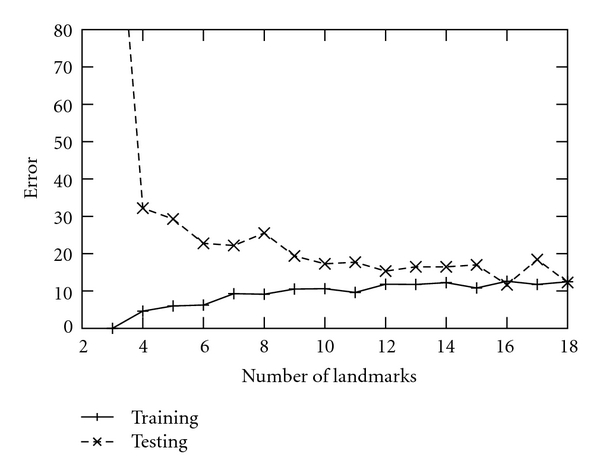
Average root mean square error after the alignment for different numbers of landmarks. Sample 20 of data set 2 (subject A) is the reference and the samples 21 to 26 are mapped to it using different numbers of landmark pairs. The results are shown separately for the training data (solid line) and test data (broken line).

For the evaluation of landmark setting, we found that 12 landmark pairs appeared to be sufficient for our test material. Figure 2 shows a representative plot of the average correction error as a function of the number of landmark pairs. Similar figures for the other cases of the data set are shown in the supporting material. The test and training set errors converge after 12 landmarks. With these LC-MALDI data, the number of mass spectra per analysis was determined by the number of MALDI spots collected, that is, just 128. With ESI-LC-MS data, however, the number of mass spectra is far greater, as the data are acquired with 20 or more full MS1 scans per minute.

**Figure 3:**
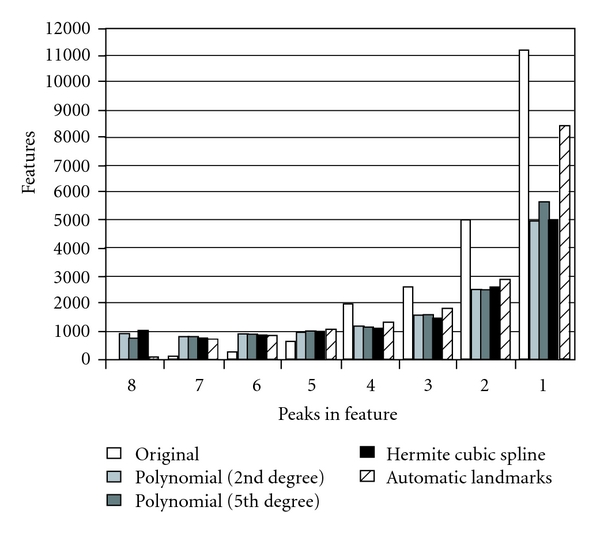
A histogram of peak matching results for a set of 8 LC-MALDI signal maps. *y*-axis gives the number of common peaks and *x*-axis shows the number of sample maps; for example, polynomial transformation (*k* = 2) found c.a 1000 peaks common in all the 8 maps.

Peak matching between data sets was tested using both LC-ESI and LC-MALDI data. Here we calculated the number of peak groups that could be matched between the maps of the test data set. We used eight LC-MALDI maps (data set 5). These maps have large differences in retention time. The maps were aligned with the MZmine embedded version of the PolyAlign and the original MZmine alignment method using a retention time tolerance of 105 seconds.  Figure 3 shows the number of peak groups that were matched between the different maps. In general, with a successful alignment technique, a large number of matching peaks would be expected from all the 8 maps. On the contrary to this, if most of the matches only occur between a few maps, the alignment may be less successful. The results of Figure 3 show that the number of peak matches clearly improved compared to the original MZmine algorithm. The Hermite transformation was the most successful method with this material, as shown by the distribution of the sizes of matching peak groups. Polynomial methods with different degrees *k* and the automatic landmarking system without any correction to landmark positions were also better than the original MZmine. We believe that the Hermite transformation demonstrated a superior performance in this application as it follows the landmarks exactly, whereas the polynomial method accepts a transformation function which does not go via the landmark points. This approach could provide a useful implementation in similar software.

**Figure 4:**
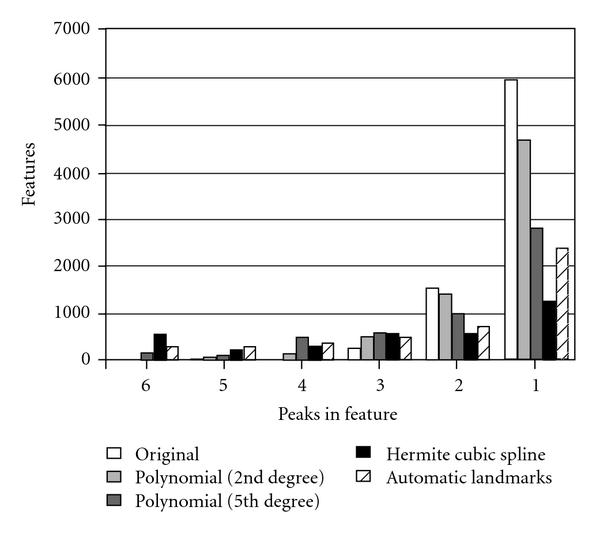
A histogram of peak matching results for a set of 6 LC-ESI signal maps. *y*-axis gives the frequencies of the found peak groups and *x*-axis shows the number of peaks in the group of matching peaks.

**Figure 5:**
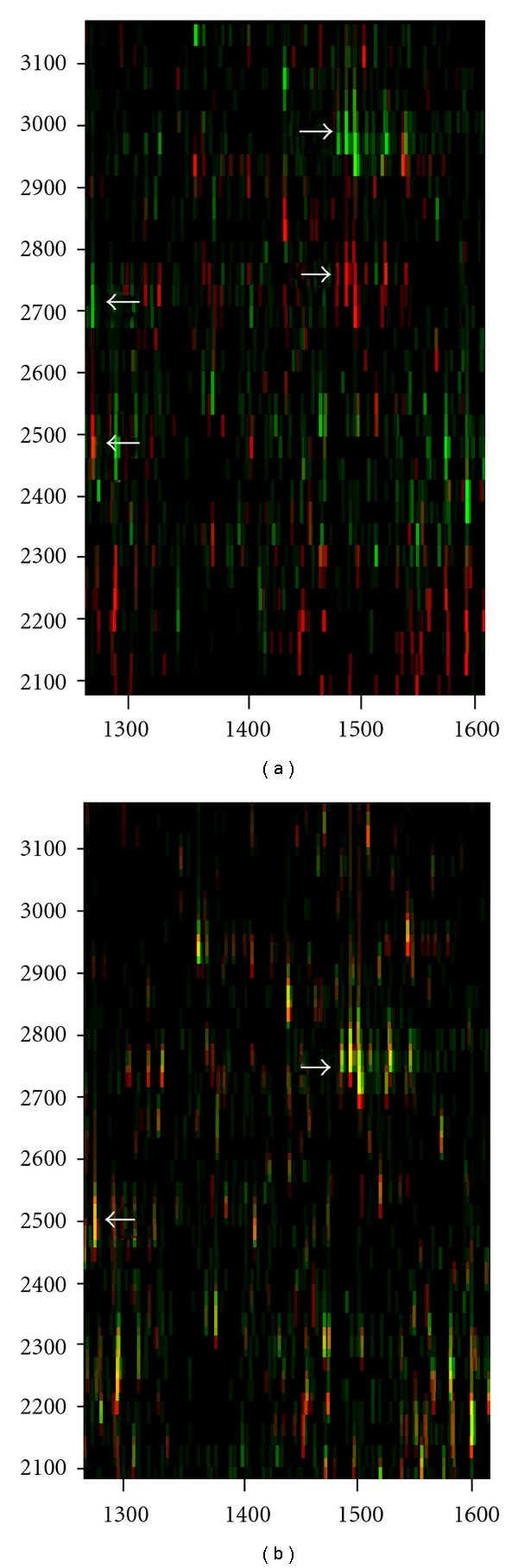
An example of alignment of two LC-MALDI maps. (a) shows two maps before alignment. The reference map is shown in red and the target in green. The target map is going to be warped. The arrows highlight two peptide peaks in the left-hand map in different locations. The other with *m*/*z* approx 1280 Da and retention time window of 2465–2535 (reference, red) and 2670–2730 (target, green) seconds. The other peak with *m*/*z* approx 1500 Da and retention time window around 2720–2810 (reference, red) and 2920–3030 (target, green) seconds. All the peaks of the target map are returned to the corresponding retention time values of the reference map in (b), and the exactly overlapping peaks turn to yellow. Since the data is from serum digest of two different individuals there is, and should be, orphan peaks that only appear in one or the other sample. Notice the longer retention time window of the higher-intensity peaks.

Figure 4 shows the corresponding results for 6 maps from the LC-ESI data set with 1% RT tolerance in matching the peaks. Again the Hermite interpolation with manual landmarking was the most successful method, but very good results were also achieved using the automatic landmarking system.  Figure 5 shows an example of a successful alignment with two LC-MALDI maps. The running time of the algorithm was a few seconds. 

In the critical review of alignment methods by Lange et al. [4] several different software packages were compared. These included MZmine, OpenMS, XAlign, XCMS, SpecArray, and msInspect, with the first three performing about equally well. Our algorithm can be used as stand-alone method to produce a set of mzXML-files for further analysis or as an enhancement of MZmine. The proposed algorithm provides an improvement over MZmine as was demonstrated in this section, so we believe that our method would be a useful tool for LC-MS alignment. Unfortunately, we were unable to use the dataset of Lange et al. for testing, as their alignment comparisons were made using a preprocessing step for feature detection, creating featureXML files. In contrast, our program was developed to directly align mzXML files and requires the full mass versus time spectral profiles that are absent from featureXML formatted data. 

## 4. Conclusions

LC-MS profiling has a great potential in comparative proteomics [26, 27]. Furthermore, the production of detailed and accountable MS profiles can be highly beneficial for biomarker discovery experiments [28] and label-free comparative quantitative proteomics [29]. The alignment of LC-MS profiles from separate measurements is an important prerequisite for such applications as it can improve data comparison and extend the depth of analysis. Although several algorithms for LC-MS data alignment are now both commercially [30] and freely [31, 32] available, in our application we aimed to provide an additional level of flexibility that would facilitate the comparison of analyses across a prolonged study whilst putting emphasis on the ease of use and user control. We have developed an image alignment-based module that employs manually selected landmarks. Although the landmark selection requires an additional level of user input, it also provides a very good visual control over the data quality. The user has full power over the alignment process, and can see the changes in the combined image pair as more landmarks are inserted and the transformation model is developed. In this phase, the user can quickly evaluate whether the data set is compromised with the insertion of erroneous landmarks, thus providing an additional level of confidence. For aiding the user the alignment module has also an option of automatic landmarking. The option includes also the possibility for easily editing the landmarks proposed by the module.

Our mzXML format-based data alignment module PolyAlign was developed for use with both LC-MALDI and electrospray data. We have created a flexible and efficient LC-MS profile alignment tool that stands out due to its real life usability. It can be operated with layman Windows user knowledge, thus saving time and effort in the early stage of MS-profiling data interpretation. This software is freely available as a stand-alone Perl-language preprocessor and as an addition to the MZmine toolbox. Both versions of the software are available together with examples of the ESI and MALDI data from the web address http://staff.cs.utu.fi/staff/jussi.salmi/Supporting.html. 

## Figures and Tables

**Table 1:** Data sets used for testing the alignment method.

Data set	Data set type	Description
1	Technical replicate (LC-MALDI)	4 technical replicates of a depleted serum sample
2	Subject A (LC-MALDI)	7 longitudinally collected depleted serum samples at different times
3	Subject B (LC-MALDI)	5 longitudinally collected depleted serum samples at different times
4	LC-ESI	6 technical replicates of a depleted serum sample
5	LC-MALDI	8 longitudinally collected depleted serum samples at different times from 2 subjects, 4 samples from each subject

**Table 2:** Root mean square errors of landmarks before and after aligning the technical replicates (data set 1). Warping polynomials of degree *k* = 1,2, 3 were applied for correcting distortion. Results for the best polynomial degrees are shown. Map 2 has been used as the reference map and the other maps were aligned to it, as indicated as target 1–4.

Target	Degree *k *	RMS error		Change	Change%
		Before	After		
1	3	54.61	6.90	−47.71	−87.4
3	2	42.15	6.65	−35.50	−84.2
4	3	30.31	13.61	−16.70	−55.1

**Table 3:** Root mean square errors of landmarks before and after aligning the patient data set for subject A (data set 2). Warping polynomials of degree *k* = 1,2, 3 were applied for correcting distortion. Results for the best polynomial degrees are shown. Map 20 has been used as the reference map and the other maps were aligned to it, as indicated as target 21–26.

Target	Degree *k *	RMS error		Change	Change%
		Before	After		
21	2	31.30	7.17	−24.13	−77.1
22	2	69.12	17.52	−51.60	−74.6
23	2	39.39	13.43	−25.96	−65.9
24	1	31.31	13.69	−17.62	−56.3
25	2	13.56	10.83	−2.73	−20.1
26	2	11.07	9.19	−1.88	−16.9

**Table 4:** Root mean square errors of landmarks before and after aligning the patient data set for subject B (data set 3). Warping polynomials of degree *k* = 1,2, 3 were applied for correcting distortion. Results for the best polynomial degrees are shown. Map 34 has been used as the reference map and the other maps were aligned to it, as indicated as target 31–35.

Target	Degree *k *	RMS error		Change	Change%
		Before	After		
31	1	22.14	16.56	−5.58	−25.3
32	1	7.46	7.10	−0.36	−4.8
33	3	31.81	9.75	−22.06	−69.3
35	3	58.91	3.45	−55.46	−94.1

## References

[1] Katajamaa M, Oresic M (2007). Data processing for mass spectrometry-based metabolomics. *Journal of Chromatography A*.

[2] Vandenbogaert M, Li-Thiao-Té S, Kaltenbach HM, Zhang R, Aittokallio T, Schwikowski B (2008). Alignment of LC-MS images, with applications to biomarker discovery and protein identification. *Proteomics*.

[3] America AHP, Cordewener JHG (2008). Comparative LC-MS: a landscape of peaks and valleys. *Proteomics*.

[4] Lange E, Tautenhahn R, Neumann S, Gröpl C (2008). Critical assessment of alignment procedures for LC-MS proteomics and metabolomics measurements. *BMC Bioinformatics*.

[5] Li XJ, Pedrioli PGA, Eng J (2004). A tool to visualize and evaluate data obtained by liquid chromatography-electrospray ionization-mass spectrometry. *Analytical Chemistry*.

[6] Wang P, Tang H, Fitzgibbon MP (2007). A statistical method for chromatographic alignment of LC-MS data. *Biostatistics*.

[7] Nielsen SB, Andersen JU, Hvelplund P, Jørgensen TJD, Sørensen M, Tomita S (2002). Triply charged bradykinin and gramicidin radical cations: their formation and the selective enhancement of charge-directed cleavage processes. *International Journal of Mass Spectrometry*.

[8] Listgarten J, Neal RM, Roweis ST, Emili A Multiple alignment of continuous time series.

[9] Listgarten J, Neal RM, Roweis ST, Wong P, Emili A (2007). Difference detection in LC-MS data for protein biomarker discovery. *Bioinformatics*.

[10] Krebs MD, Tingley RD, Zeskind JE, Holmboe ME, Kang JM, Davis CE (2006). Alignment of gas chromatography-mass spectrometry data by landmark selection from complex chemical mixtures. *Chemometrics and Intelligent Laboratory Systems*.

[11] Prakash A, Mallick P, Whiteaker J (2006). Signal maps for mass spectrometry-based comparative proteomics. *Molecular and Cellular Proteomics*.

[12] Jaitly N, Monroe ME, Petyuk VA, Clauss TRW, Adkins JN, Smith RD (2006). Robust algorithm for alignment of liquid chromatography-mass spectrometry analyses in an accurate mass and time tag data analysis pipeline. *Analytical Chemistry*.

[13] Mirgorodskaya E, Braeuer C, Fucini P, Lehrach H, Gobom J (2005). Nanoflow liquid chromatography coupled to matrix-assisted laser desorption/ionization mass spectrometry: sample preparation, data analysis, and application to the analysis of complex peptide mixtures. *Proteomics*.

[14] Hattan SJ, Parker KC (2006). Methodology utilizing MS signal intensity and LC retention time for quantitative analysis and precursor ion selection in proteomic LC-MALDI analyses. *Analytical Chemistry*.

[15] Neubert H, Bonnert TP, Rumpel K, Hunt BT, Henle ES, Lames IT (2008). Label-Free detection of differential protein expression by LC/MALDI mass spectrometry. *Journal of Proteome Research*.

[16] Katajamaa M, Miettinen J, Oresic M (2006). MZmine: toolbox for processing and visualization of mass spectrometry based molecular profile data. *Bioinformatics*.

[17] Kupila A, Muona P, Simell T (2001). Juvenile diabetes research foundation centre for the prevention of type I diabetes in Finland. *Diabetologia*.

[18] Bylund D, Danielsson R, Malmquist G, Markides KE (2002). Chromatographic alignment by warping and dynamic programming as a pre-processing tool for PARAFAC modelling of liquid chromatography-mass spectrometry data. *Journal of Chromatography A*.

[19] Cormen TH, Leiserson CE, Rivest RL (1990). *Introduction to Algorithms*.

[20] Prince JT, Marcotte EM (2006). Chromatographic alignment of ESI-LC-MS proteomics data sets by ordered bijective interpolated warping. *Analytical Chemistry*.

[21] Kocić LM, Milovanović GV (1997). Shape preserving approximations by polynomials and splines. *Computers and Mathematics with Applications*.

[22] Samet H (1984). The quadtree and related hierarchical data structures. *Computing Surveys*.

[23] Katajamaa M, Oresic M (2005). Processing methods for differential analysis of LC/MS profile data. *BMC Bioinformatics*.

[24] Oresic M, Simell S, Sysi-Aho M (2008). Dysregulation of lipid and amino acid metabolism precedes islet autoimmunity in children who later progress to type 1 diabetes. *Journal of Experimental Medicine*.

[25] Dekker LJ, Dalebout JC, Siccama I, Jenster G, Smitt PAS, Luider TM (2005). A new method to analyze matrix-assisted laser desorption/ionization time-of-flight peptide profiling mass spectra. *Rapid Communications in Mass Spectrometry*.

[26] Mueller LN, Rinner O, Schmidt A (2007). SuperHirn—a novel tool for high resolution LC-MS-based peptide/protein profiling. *Proteomics*.

[27] Schmidt A, Gehlenborg N, Bodenmiller B (2008). An integrated, directed mass spectrometric approach for in-depth characterization of complex peptide mixtures. *Molecular and Cellular Proteomics*.

[28] Jacobs JM, Adkins JN, Qian WJ (2005). Utilizing human blood plasma for proteomic biomarker discovery. *Journal of Proteome Research*.

[29] May D, Fitzgibbon M, Liu Y (2007). A platform for accurate mass and time analyses of mass spectrometry data. *Journal of Proteome Research*.

[30] Kaplan A, Söderström M, Fenyö D (2007). An automated method for scanning LC-MS data sets for significant peptides and proteins, including quantitative profiling and interactive confirmation. *Journal of Proteome Research*.

[31] Suits F, Lepre J, Du P, Bischoff R, Horvatovich P (2008). Two-dimensional method for time aligning liquid chromatography-mass spectrometry data. *Analytical Chemistry*.

[32] Hwang D, Zhang N, Lee H (2008). MS-BID: a Java package for label-free LC-MS-based comparative proteomic analysis. *Bioinformatics*.

